# Concluding remarks

**DOI:** 10.1186/1471-2393-12-S1-A14

**Published:** 2012-08-28

**Authors:** Edwin A Mitchell

**Affiliations:** 1 University of Auckland, New Zealand

## 

**Figure 1:**
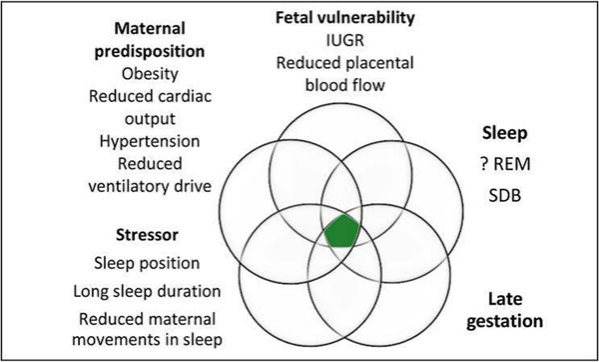
Conceptual model for the mechanism of stillbirths (developed by EM).

**Figure 2:**
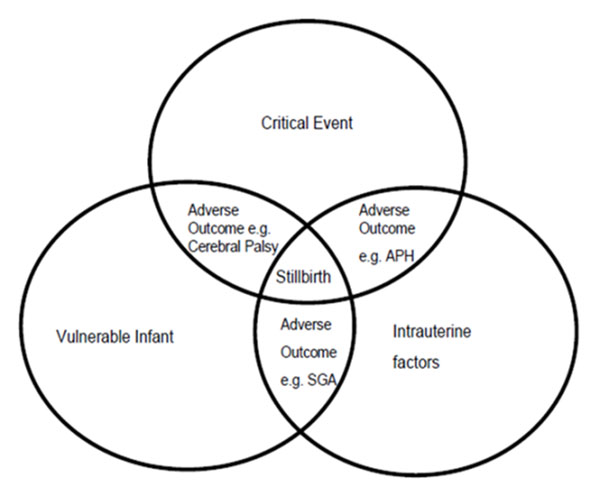
Conceptual model for the mechanism of stillbirths (developed by JW) [2] and adapted from Rognum and Saugstad [3].

This forum allowed for the discussion and exploration of a number of issues relating to stillbirth that have either been given minimal attention, or are emerging hypotheses. Research has identified many risk factors that may contribute or are associated with stillbirth. These risk factors have odds ratios between 2 and 3 indicating that it is unlikely that any of these are the definitive cause of stillbirth, rather they might be additive or interact together resulting in a stillbirth particularly if the fetus is somehow vulnerable. Two of the authors (EM & JW) presented conceptual models at the conference which were adapted from the SIDS triple risk model (Figure 1 and 2 respectively) [1-3]. Both models are more applicable to unexplained stillbirths rather than when there is a single clear cut cause. These show that that stillbirth may occur when there are prevailing risk factors, especially intrauterine or maternal disposition, accompanied by a stressor or critical event if the fetus is vulnerable.

These conceptual models were endorsed by the researchers, and suggestions for improving them were made, including adding specific risk factors such as maternal diabetes, placental abruption, maternal smoking and maternal age.

There was healthy and robust debate, both between the presenters and the attendees and between the researchers themselves, which brought a richness to the meeting. Alongside the energy and passion to see a real change in the devastating number of babies that die before birth, there was a moderating voice from a number of the researchers for the need for robust, peer reviewed evidence to be generated before significant recommendations for change in practice, or public health campaigns are launched.
